# Supplementary material for: Tuning of the Electrostatic Potentials on the Surface of the Sulfur Atom in Organic Molecules: Theoretical Design and Experimental Assessment
Source: Molecules. 2023 May 6;28(9):3919. doi: 10.3390/molecules28093919 (PMC10180200; doi:10.3390/molecules28093919)
Supplement: Supplementary file 1 [file molecules-28-03919-s001.zip › molecules-2342883-supplementary/File S1.docx]

Cartesian coordinates of 2-BPTD:

S 0.000003 -0.860425 -0.000074

N 0.674114 1.595347 0.000085

N -0.674126 1.595360 0.000056

N 2.966677 -1.168209 0.000517

N -2.966673 -1.168207 -0.000440

C 1.173711 0.394899 -0.000025

C -1.173713 0.394906 0.000035

C 2.616773 0.116893 -0.000037

C 3.537280 1.161776 -0.000492

H 3.182736 2.182073 -0.000853

C 4.882936 0.842362 -0.000471

H 5.629925 1.624720 -0.000881

C 5.258313 -0.493037 0.000064

H 6.298693 -0.785042 0.000069

C 4.261556 -1.458881 0.000580

H 4.514616 -2.512434 0.000982

C -2.616772 0.116881 0.000040

C -3.537279 1.161781 0.000440

H -3.182717 2.182073 0.000773

C -4.882923 0.842369 0.000404

H -5.629929 1.624712 0.000757

C -5.258308 -0.493046 -0.000071

H -6.298696 -0.785013 -0.000103

C -4.261573 -1.458885 -0.000505

H -4.514620 -2.512434 -0.000860

Cartesian coordinates of TD:

S 1.166747 -0.000083 -0.000023

N -1.277721 0.679896 -0.000485

N -1.277770 -0.679519 0.000430

C -0.083781 1.173031 0.000421

C -0.084063 -1.173195 -0.000289

H 0.114377 2.233034 -0.000160

H 0.113181 -2.233363 0.000128

Cartesian coordinates of 3-BPTD:

S -0.000075 0.930728 -0.000092

N -4.841079 -1.006994 -0.000377

N -0.673363 -1.512343 0.000638

N 0.673417 -1.512338 0.000579

N 4.841108 -1.006964 -0.000513

C -3.524040 -1.149134 -0.000176

H -3.132827 -2.158643 -0.000092

C -2.634539 -0.070066 0.000133

C -3.168104 1.213208 0.000171

H -2.520326 2.081563 0.000416

C -4.542658 1.367391 -0.000099

H -4.994295 2.348933 -0.000118

C -5.335441 0.228756 -0.000369

H -6.415866 0.312253 -0.000630

C -1.191809 -0.322616 0.000378

C 1.191816 -0.322619 0.000194

C 2.634536 -0.070079 0.000039

C 3.168077 1.213154 0.000225

H 2.520401 2.081592 0.000619

C 4.542661 1.367399 0.000077

H 4.994191 2.348989 0.000274

C 5.335465 0.228840 -0.000266

H 6.415881 0.312307 -0.000392

C 3.524125 -1.149151 -0.000366

H 3.132931 -2.158662 -0.000552

Cartesian coordinates of 1,4-DITFB:

C 0.000000 1.189780 0.692494

C 0.000000 0.000000 1.402362

C 0.000000 -1.189780 0.692494

C 0.000000 -1.189780 -0.692494

C 0.000000 0.000000 -1.402362

C 0.000000 1.189780 -0.692494

F 0.000000 2.355185 -1.325553

F 0.000000 2.355185 1.325553

F 0.000000 -2.355185 1.325553

F 0.000000 -2.355185 -1.325553

I 0.000000 0.000000 3.469089

I 0.000000 0.000000 -3.469089

Cartesian coordinates of 1,3,5-TFTIB:

C 0.000000 1.371915 0.000000

C -1.212872 0.700252 0.000000

C -1.188114 -0.685958 0.000000

C 0.000000 -1.400504 0.000000

C 1.188114 -0.685958 0.000000

C 1.212872 0.700252 0.000000

F 0.000000 2.696603 0.000000

F -2.335327 -1.348302 0.000000

F 2.335327 -1.348302 0.000000

I -3.005623 1.735297 0.000000

I 3.005623 1.735297 0.000000

I 0.000000 -3.470594 0.000000
