# Supplementary material for: Tuning of the Electrostatic Potentials on the Surface of the Sulfur Atom in Organic Molecules: Theoretical Design and Experimental Assessment
Source: Molecules. 2023 May 6;28(9):3919. doi: 10.3390/molecules28093919 (PMC10180200; doi:10.3390/molecules28093919)
Supplement: Supplementary file 1 [file molecules-28-03919-s001.zip › molecules-2342883-supplementary/File S2.docx]

Cartesian coordinates of 2-BPTD···1,4-DITFB:

I 0.262736 -1.317912 0.312930

I -6.321712 0.860800 -0.284073

F -1.068404 1.059316 -1.324883

F -3.581983 1.938947 -1.524822

F -4.980698 -1.468159 1.413554

F -2.480204 -2.328116 1.623015

C -1.710561 -0.628284 0.170155

C -2.018044 0.444604 -0.626199

C -3.333549 0.878893 -0.737102

C -4.352866 0.270724 -0.062941

C -4.026011 -0.818712 0.739405

C -2.726855 -1.254714 0.862047

S 3.524859 0.353685 0.183281

N 5.823481 -0.428122 -0.567341

N 5.931600 0.946757 -0.418648

N 3.043461 -2.617034 0.001450

N 3.376966 3.284166 0.497092

C 4.644516 -0.868193 -0.289971

C 4.808497 1.469540 -0.036740

C 4.297156 -2.290756 -0.357128

C 5.232176 -3.222676 -0.760322

H 6.221065 -2.895172 -1.044732

C 4.851751 -4.551083 -0.793678

H 5.562612 -5.303084 -1.110741

C 3.593312 -4.910480 -0.402074

H 3.271259 -5.941867 -0.402397

C 2.710940 -3.909974 -0.032423

H 1.693999 -4.145128 0.259841

C 4.613285 2.911636 0.195460

C 5.701039 3.779044 0.094183

H 6.676470 3.386934 -0.151593

C 5.460979 5.114193 0.329242

H 6.268395 5.832317 0.270912

C 4.186938 5.520932 0.621668

H 3.959345 6.562217 0.802138

C 3.180799 4.578305 0.703297

H 2.166908 4.875517 0.943697

Cartesian coordinates of 3,5-dichloropyridine···1,4-DITFB:

I 0.26273600 -1.31791200 0.31293000

I -6.32171200 0.86080000 -0.28407300

F -1.06840400 1.05931600 -1.32488300

F -3.58198300 1.93894700 -1.52482200

F -4.98069800 -1.46815900 1.41355400

F -2.48020400 -2.32811600 1.62301500

C -1.71056100 -0.62828400 0.17015500

C -2.01804400 0.44460400 -0.62619900

C -3.33354900 0.87889300 -0.73710200

C -4.35286600 0.27072400 -0.06294100

C -4.02601100 -0.81871200 0.73940500

C -2.72685500 -1.25471400 0.86204700

N 3.04346100 -2.61703400 0.00145000

C 4.29715600 -2.29075600 -0.35712800

C 5.23217600 -3.22267600 -0.76032200

C 4.85175100 -4.55108300 -0.79367800

H 5.56261200 -5.30308400 -1.11074100

C 3.59331200 -4.91048000 -0.40207400

C 2.71094000 -3.90997400 -0.03242300

H 1.69399900 -4.14512800 0.25984100

Cl 6.84394109 -2.68888554 -1.22387464

Cl 3.06872711 -6.59048310 -0.40260013

H 4.55070393 -1.25238684 -0.30810820

Cartesian coordinates of pyridine···1,4-DITFB:

I 0.26273600 -1.31791200 0.31293000

I -6.32171200 0.86080000 -0.28407300

F -1.06840400 1.05931600 -1.32488300

F -3.58198300 1.93894700 -1.52482200

F -4.98069800 -1.46815900 1.41355400

F -2.48020400 -2.32811600 1.62301500

C -1.71056100 -0.62828400 0.17015500

C -2.01804400 0.44460400 -0.62619900

C -3.33354900 0.87889300 -0.73710200

C -4.35286600 0.27072400 -0.06294100

C -4.02601100 -0.81871200 0.73940500

C -2.72685500 -1.25471400 0.86204700

N 3.04346100 -2.61703400 0.00145000

C 4.29715600 -2.29075600 -0.35712800

C 5.23217600 -3.22267600 -0.76032200

H 6.22106500 -2.89517200 -1.04473200

C 4.85175100 -4.55108300 -0.79367800

H 5.56261200 -5.30308400 -1.11074100

C 3.59331200 -4.91048000 -0.40207400

H 3.27125900 -5.94186700 -0.40239700

C 2.71094000 -3.90997400 -0.03242300

H 1.69399900 -4.14512800 0.25984100

H 4.55070393 -1.25238684 -0.30810820
