# Supplementary material for: Tuning of the Electrostatic Potentials on the Surface of the Sulfur Atom in Organic Molecules: Theoretical Design and Experimental Assessment
Source: Molecules. 2023 May 6;28(9):3919. doi: 10.3390/molecules28093919 (PMC10180200; doi:10.3390/molecules28093919)

checkCIF/PLATON report

Structure factors have been supplied for datablock(s) 2162326

THIS REPORT IS FOR GUIDANCE ONLY. IF USED AS PART OF A REVIEW PROCEDURE FOR PUBLICATION, IT SHOULD NOT REPLACE THE EXPERTISE OF AN EXPERIENCED CRYSTALLOGRAPHIC REFEREE.

No syntax errors found. CIF dictionary Interpreting this report

Datablock: 2162326

```
Bond precision:   C-C = 0.0107 A          Wavelength=0.71073

Cell:             a=22.0520(5)     b=7.84693(17)      c=11.8297(2)
                  alpha=90         beta=99.301(2)     gamma=90
Temperature:      293 K

                 Calculated                    Reported
Volume           2020.11(7)                    2020.10(8)
Space group      C c                           C 1 c 1
Hall group       C -2yc                        C -2yc
Moiety formula   C12 H8 N4 S, C6 F4 I2         C6 F4 I2, C12 H8 N4 S
Sum formula      C18 H8 F4 I2 N4 S             C18 H8 F4 I2 N4 S
Mr               642.14                        642.14
Dx,g cm-3        2.111                         2.111
Z                4                             4
Mu (mm-1)        3.265                         3.265
F000             1208.0                        1208.0
F000'            1205.26
h,k,lmax         26,9,14                       26,9,14
Nref             3550[ 1777]                   3542
Tmin,Tmax        0.403,0.504                   0.579,1.000
Tmin'            0.373

Correction method= # Reported T Limits: Tmin=0.579 Tmax=1.000
AbsCorr = MULTI-SCAN

Data completeness= 1.99/1.00        Theta(max)= 24.997

R(reflections)= 0.0224( 3436)                          wR2(reflections)=
                                                       0.0529( 3542)

S = 1.074                  Npar= 263
```

The following ALERTS were generated. Each ALERT has the format
test-name_ALERT_alert-type_alert-level.
Click on the hyperlinks for more details of the test.

Alert level C

```
PLAT042_ALERT_1_C Calc. and Reported MoietyFormula Strings  Differ     Please Check
PLAT090_ALERT_3_C Poor Data / Parameter Ratio (Zmax > 18) ........       6.75 Note
PLAT342_ALERT_3_C Low Bond Precision on  C-C Bonds ..............    0.01069 Ang.
```

Alert level G

```
PLAT012_ALERT_1_G N.O.K.   _shelx_res_checksum Found in CIF ......     Please Check
PLAT013_ALERT_1_G N.O.K.   _shelx_hkl_checksum Found in CIF ......     Please Check
PLAT199_ALERT_1_G Reported _cell_measurement_temperature ..... (K)        293 Check
PLAT200_ALERT_1_G Reported   _diffrn_ambient_temperature ..... (K)        293 Check
PLAT431_ALERT_2_G Short Inter HL..A Contact  I1       ..N3       .       3.09 Ang.
                                                      x,y,z  =      1_555 Check
PLAT431_ALERT_2_G Short Inter HL..A Contact  I2       ..N1       .       3.24 Ang.
                                           1/2+x,-1/2+y,1+z  =      3_546 Check
PLAT431_ALERT_2_G Short Inter HL..A Contact  I2       ..N2       .       3.24 Ang.
                                           1/2+x,-1/2+y,1+z  =      3_546 Check
PLAT883_ALERT_1_G No Info/Value for _atom_sites_solution_primary .     Please Do !
PLAT909_ALERT_3_G Percentage of I>2sig(I) Data at Theta(Max) Still        90% Note
PLAT910_ALERT_3_G Missing # of FCF Reflection(s) Below Theta(Min).          3 Note
PLAT961_ALERT_5_G Dataset Contains no Negative Intensities .......     Please Check
PLAT967_ALERT_5_G Note: Two-Theta Cutoff Value in Embedded .res ..       50.0 Degree
PLAT978_ALERT_2_G Number C-C Bonds with Positive Residual Density.          0 Info
```

0 **ALERT level A** = Most likely a serious problem - resolve or explain
0 **ALERT level B** = A potentially serious problem, consider carefully
3 **ALERT level C** = Check. Ensure it is not caused by an omission or oversight
13 **ALERT level G** = General information/check it is not something unexpected

6 ALERT type 1 CIF construction/syntax error, inconsistent or missing data
4 ALERT type 2 Indicator that the structure model may be wrong or deficient
4 ALERT type 3 Indicator that the structure quality may be low
0 ALERT type 4 Improvement, methodology, query or suggestion
2 ALERT type 5 Informative message, check

It is advisable to attempt to resolve as many as possible of the alerts in all categories. Often the minor alerts point to easily fixed oversights, errors and omissions in your CIF or refinement strategy, so attention to these fine details can be worthwhile. In order to resolve some of the more serious problems it may be necessary to carry out additional measurements or structure refinements. However, the purpose of your study may justify the reported deviations and the more serious of these should normally be commented upon in the discussion or experimental section of a paper or in the "special_details" fields of the CIF. checkCIF was carefully designed to identify outliers and unusual parameters, but every test has its limitations and alerts that are not important in a particular case may appear. Conversely, the absence of alerts does not guarantee there are no aspects of the results needing attention. It is up to the individual to critically assess their own results and, if necessary, seek expert advice.

Publication of your CIF in IUCr journals

A basic structural check has been run on your CIF. These basic checks will be run on all CIFs submitted for publication in IUCr journals (*Acta Crystallographica*, *Journal of Applied Crystallography*, *Journal of Synchrotron Radiation*); however, if you intend to submit to *Acta Crystallographica Section C* or *E* or *IUCrData*, you should make sure that full publication checks are run on the final version of your CIF prior to submission.

Publication of your CIF in other journals

Please refer to the *Notes for Authors* of the relevant journal for any special instructions relating to CIF submission.

PLATON version of 28/11/2022; check.def file version of 28/11/2022

Datablock 2162326 - ellipsoid plot

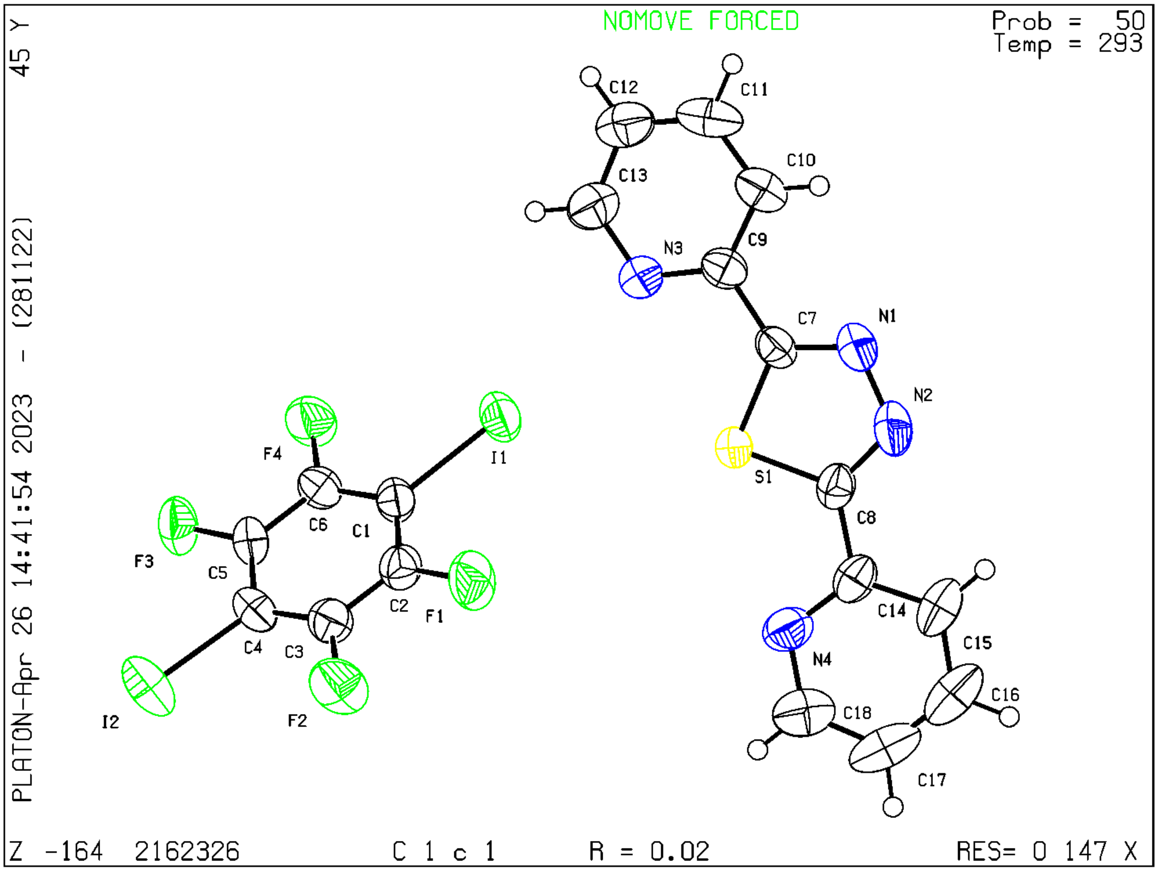

checkCIF/PLATON report

Structure factors have been supplied for datablock(s) 2162328

THIS REPORT IS FOR GUIDANCE ONLY. IF USED AS PART OF A REVIEW PROCEDURE FOR PUBLICATION, IT SHOULD NOT REPLACE THE EXPERTISE OF AN EXPERIENCED CRYSTALLOGRAPHIC REFEREE.

No syntax errors found. CIF dictionary Interpreting this report

Datablock: 2162328

```
Bond precision:   C-C = 0.0062 A          Wavelength=0.71073

Cell:             a=7.5248(2)     b=19.6708(5)        c=14.6179(5)
                  alpha=90        beta=103.947(3)     gamma=90
Temperature:      293 K

                Calculated                    Reported
Volume          2099.94(11)                   2099.94(11)
Space group     P 21/n                        P 1 21/n 1
Hall group      -P 2yn                        -P 2yn
Moiety formula  C12 H8 N4 S, C6 F3 I3         C6 F3 I3, C12 H8 N4 S
Sum formula     C18 H8 F3 I3 N4 S             C18 H8 F3 I3 N4 S
Mr              750.04                        750.04
Dx,g cm-3       2.372                         2.372
Z               4                             4
Mu (mm-1)       4.603                         4.603
F000            1384.0                        1384.0
F000'           1379.39
h,k,lmax        9,23,17                       9,23,17
Nref            3906                          3854
Tmin,Tmax       0.262,0.576                   0.487,1.000
Tmin'           0.220

Correction method= # Reported T Limits: Tmin=0.487 Tmax=1.000
AbsCorr = MULTI-SCAN

Data completeness= 0.987           Theta(max)= 25.498

R(reflections)= 0.0327( 3366)                         wR2(reflections)=
                                                      0.0699( 3854)
S = 1.047                   Npar= 263
```

The following ALERTS were generated. Each ALERT has the format
test-name_ALERT_alert-type_alert-level.
Click on the hyperlinks for more details of the test.

Alert level C

```
PLAT042_ALERT_1_C Calc. and Reported MoietyFormula Strings  Differ     Please Check
PLAT906_ALERT_3_C Large K Value in the Analysis of Variance ......      3.337 Check
PLAT910_ALERT_3_C Missing # of FCF Reflection(s) Below Theta(Min).          8 Note
PLAT911_ALERT_3_C Missing FCF Refl Between Thmin & STh/L=    0.600         20 Report
PLAT975_ALERT_2_C Check Calcd Resid. Dens.  1.05Ang From N2      .       0.52 eA-3
```

Alert level G

```
PLAT012_ALERT_1_G N.O.K.   _shelx_res_checksum Found in CIF ......     Please Check
PLAT013_ALERT_1_G N.O.K.   _shelx_hkl_checksum Found in CIF ......     Please Check
PLAT199_ALERT_1_G Reported _cell_measurement_temperature ..... (K)        293 Check
PLAT200_ALERT_1_G Reported   _diffrn_ambient_temperature ..... (K)        293 Check
PLAT431_ALERT_2_G Short Inter HL..A Contact  I1       ..N1       .       2.91 Ang.
                                          3/2-x,1/2+y,1/2-z  =      2_655 Check
PLAT431_ALERT_2_G Short Inter HL..A Contact  I2       ..N4       .       2.90 Ang.
                                         3/2-x,-1/2+y,1/2-z  =      2_645 Check
PLAT431_ALERT_2_G Short Inter HL..A Contact  I3       ..N2       .       3.04 Ang.
                                         1/2+x,3/2-y,-1/2+z  =      4_675 Check
PLAT431_ALERT_2_G Short Inter HL..A Contact  I3       ..N3       .       3.33 Ang.
                                         1/2+x,3/2-y,-1/2+z  =      4_675 Check
PLAT883_ALERT_1_G No Info/Value for _atom_sites_solution_primary .     Please Do !
PLAT912_ALERT_4_G Missing # of FCF Reflections Above STh/L=  0.600         24 Note
PLAT941_ALERT_3_G Average HKL Measurement Multiplicity ..........         2.9 Low
PLAT961_ALERT_5_G Dataset Contains no Negative Intensities .......     Please Check
PLAT967_ALERT_5_G Note: Two-Theta Cutoff Value in Embedded .res ..       51.0 Degree
PLAT978_ALERT_2_G Number C-C Bonds with Positive Residual Density.          1 Info
```

0 **ALERT level A** = Most likely a serious problem - resolve or explain
0 **ALERT level B** = A potentially serious problem, consider carefully
5 **ALERT level C** = Check. Ensure it is not caused by an omission or oversight
14 **ALERT level G** = General information/check it is not something unexpected

6 ALERT type 1 CIF construction/syntax error, inconsistent or missing data
6 ALERT type 2 Indicator that the structure model may be wrong or deficient
4 ALERT type 3 Indicator that the structure quality may be low
1 ALERT type 4 Improvement, methodology, query or suggestion
2 ALERT type 5 Informative message, check

It is advisable to attempt to resolve as many as possible of the alerts in all categories. Often the minor alerts point to easily fixed oversights, errors and omissions in your CIF or refinement strategy, so attention to these fine details can be worthwhile. In order to resolve some of the more serious problems it may be necessary to carry out additional measurements or structure refinements. However, the purpose of your study may justify the reported deviations and the more serious of these should normally be commented upon in the discussion or experimental section of a paper or in the "special_details" fields of the CIF. checkCIF was carefully designed to identify outliers and unusual parameters, but every test has its limitations and alerts that are not important in a particular case may appear. Conversely, the absence of alerts does not guarantee there are no aspects of the results needing attention. It is up to the individual to critically assess their own results and, if necessary, seek expert advice.

Publication of your CIF in IUCr journals

A basic structural check has been run on your CIF. These basic checks will be run on all CIFs submitted for publication in IUCr journals (*Acta Crystallographica*, *Journal of Applied Crystallography*, *Journal of Synchrotron Radiation*); however, if you intend to submit to *Acta Crystallographica Section C* or *E* or *IUCrData*, you should make sure that full publication checks are run on the final version of your CIF prior to submission.

Publication of your CIF in other journals

Please refer to the *Notes for Authors* of the relevant journal for any special instructions relating to CIF submission.

PLATON version of 28/11/2022; check.def file version of 28/11/2022

Datablock 2162328 - ellipsoid plot

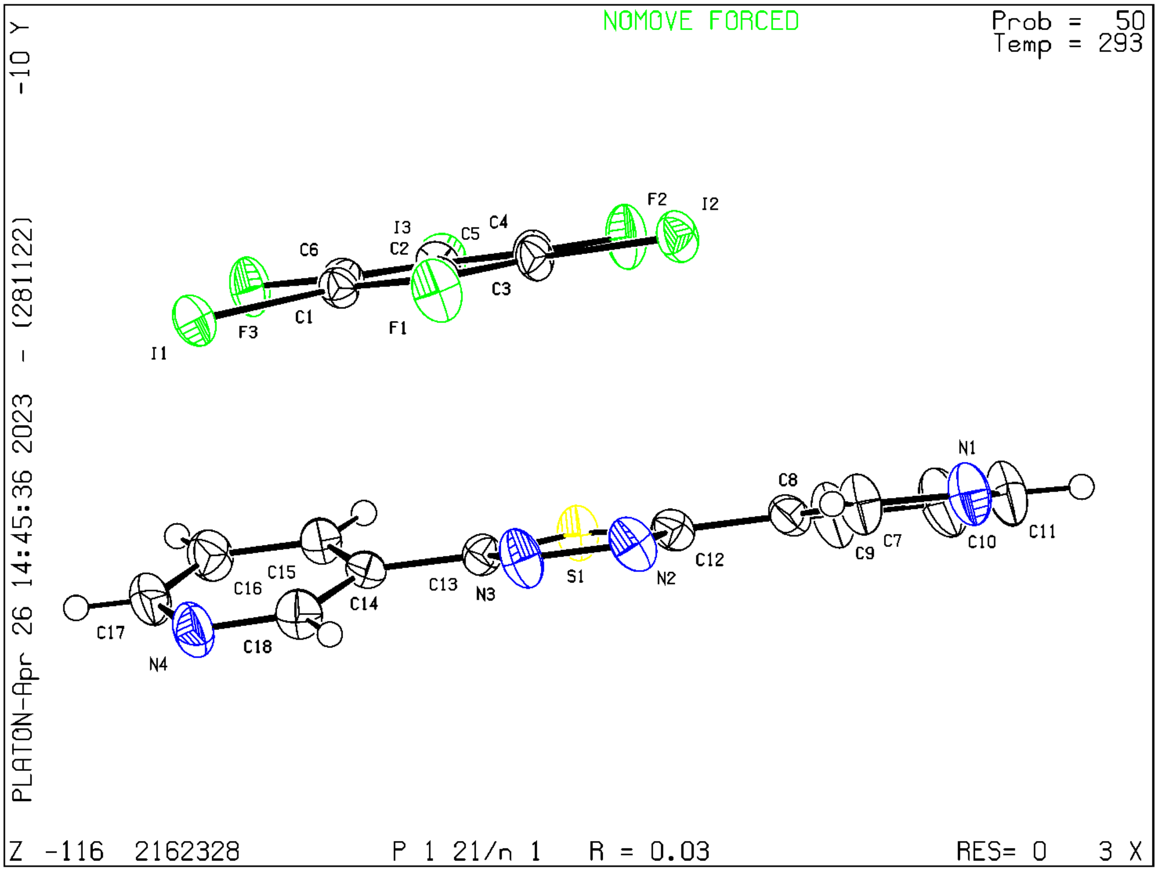

Supplement: Supplementary file 1 [file molecules-28-03919-s001.zip › molecules-2342883-supplementary/File S3.pdf]
